# Population-based retrospective cohort study on community-acquired pneumonia hospitalization in children with a ventricular septal defect

**DOI:** 10.1038/s41598-024-59510-9

**Published:** 2024-04-23

**Authors:** Myongsoon Sung, Ju Hee Kim, Eun Kyo Ha, Jeewon Shin, Ji Hee Kwak, Hye Mi Jee, Man Yong Han

**Affiliations:** 1 Department of Pediatrics, Soonchunhyang University Gumi Hospital, Gumi, Republic of Korea; 2 Department of Pediatrics, National Jewish Health, Denver, CO USA; 3 Department of Pediatrics, Kyung Hee University College of Medicine, Seoul, Korea; 4 Department of Pediatrics, Hallym University Kangnam Sacred Heart Hospital, Seoul, Korea; 5 Department of Pediatrics, CHA Bundang Medical Center, CHA University School of Medicine, 351 Yatap-Dong, Bundang-Gu, Seongnam, 463-712 Gyonggi-Do Korea; 6 Department of Pediatrics, Kangbuk Samsung Hospital, Sungkyunkwan University School of Medicine, Seoul, Korea

**Keywords:** Cardiology, Health care, Medical research, Risk factors

## Abstract

The cohort consisted of 9400 exposed children diagnosed with ventricular septal defect (VSD). The risk of community-acquired pneumonia (CAP) or asthma with VSD was assessed using the Cox proportional hazard model with an inverse probability of treatment weighting. During a mean follow-up of 6.67 years (starting from 12 months after birth), there were 2100 CAP admission cases among exposed patients (incidence rate: 33.2 per 1000 person-years) and 20,109 CAP admission cases among unexposed children (incidence rate: 29.6 per 1000 person-years), with hazard ration of 1.09 (95% CI 1.04–1.14).

## Introduction

Community-acquired pneumonia (CAP) is one of the most common infections in the pediatric population and the most frequent reason for hospitalization^1,2^. It is well known that the leading cause of CAP is a viral infection^1,2^. Also, patients with ventricular septal defect (VSD) have a higher risk for complications with viral diseases^3,4^.

A VSD is a defect in the septum separating the two lower chambers of the heart ventricles and is the most common congenital heart malformation^5,6^. Small VSDs often close independently, and if the opening is small such as pin size, the heart and lungs do not have to work harder^7^. Meanwhile, VSD with congenital heart failure is a common cyanotic congenital heart disease (CHD) in childhood predisposing to bronchopneumonia^8–10^. CHD increases pulmonary blood flow and is a common predisposing factor for pneumonia in children^11^. Pulmonary edema of VSD patients can lead to congestive heart failure and become a nidus of infection for lower respiratory tract infection^3,4,12^.

Moreover, as with CAP, asthma in children is a significant concern because it increases the number of hospital visits and has been an economic burden more than asthma in adults^13,14^. In particular, asthma medication costs were found to be the largest cost factor in children ($382.09 or 41.3% of total direct cost), whilst they were reported as being lower in adults^13^. A few studies have suggested that asthma or airway hypersensitivity is more common in children with CHD than in the general population^15–17^.

Based on these results, we assumed that VSD might have some associations with the severity of CAP and asthma in the pediatric population during childhood. However, recently, a few reports have suggested a surge in the risk of hospitalization existence of CAP and asthma in children with VSD^18^. Thus, the present study aimed to use an extensive national population-based data set that included stratified questionnaires and information on healthcare utilization to determine the association of VSD with risks of CAP and asthma hospitalization in children. Furthermore, we thoroughly analyzed detailed information regarding family income, residency, and birth history.

## Methods

### Data source

Demographic characteristics, health care utilization, and outcome data of all participants were obtained from the National Health Insurance Service (NHIS) of Korea and the National Health Screening Program for Infants and Children (NHSPIC) database. All residents must participate in the NHIS. It maintains health records regarding healthcare utilization, prescriptions, and national health screening programs for all residents of Korea. Also, before the NHIS of Korea and the NHSPIC, all residents agreed to provide their records to investigators and sign in. The NHSPIC database includes information about developmental screening implemented from the second to the seventh round for children aged 9–71 months.

### Ethics approval and consent to participate

Conducted in alignment with the Declaration of Helsinki, the Korea National Institute for Bioethics Policy Institutional Review Board (IRB) approved this study (P01-201603–21-005) due to its retrospective nature. The Korea National Institute for Bioethics Policy IRB approved that all methods in this study were performed according to the relevant guidelines and regulations. Also, because of its retrospective nature, the Korea National Institute for Bioethics Policy Institutional Review Board waived informed consent.

### Study population and study design

**Figure 1:**
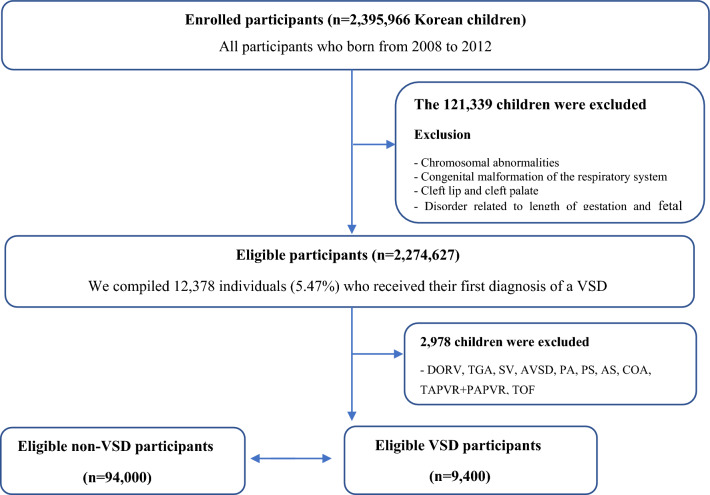
Disposition of children in the study cohort. Individuals were identified from the National Health Insurance Service (NHIS) of Korea and the National Health Screening Program for Infants and Children (NHSPIC) database. After 1:10 random sampling, 9,400 children were assigned to the VSD group and 94,000 children were assigned to the non-VSD group. *DORV* double outlet right ventricle, *AS* aortic stenosis, *ASD* atrial septal defect, *AVSD* atrioventricular septal defect, *COA* coarctation of the aorta, *PA* pulmonary atresia, *PAPVR* partial anomalous pulmonary venous return, *PAS* pulmonary artery stenosis, *SV* single ventricle, *TAPVR* total anomalous pulmonary venous return, *TOF* tetralogy of Fallot, *VSD* ventricle septal defect.

Figure 1 shows the study population’s selection. The present study included 2,395,966 Korean children born between 2008 and 2012. Of these, 121,339 were excluded according to the exclusion criteria (chromosomal abnormalities, congenital malformation of the respiratory system, cleft lip and cleft palate, disorder related to gestation length and fetal growth), leaving 2,274,627 eligible patients for further analyses. The 12,378 individuals received their first diagnosis of a VSD between birth and December 31, 2019. Of them, 2978 children have VSD with complex CHD (ex. Double outlet right ventricle (DORV), transposition of the great arteries (TGA), single ventricle (SV), atrioventricular septal defect (AVSD), pulmonary atresia (PA), pulmonary artery stenosis (PAS), aortic stenosis (AS), coarctation of the aorta (COA), total anomalous pulmonary venous return (TAPVR) plus partial anomalous pulmonary venous return (PAPVR), or tetralogy of Fallot (TOF) were excluded. Finally, 9400 children were assigned to the VSD group. After a 1:10 complete random sampling, 94,000 children were in the non-VSD group. Also, the VSD group was divided into two subgroups: VAD with heart failure (HF) (N = 513) and VAD without HF(N = 8,887).

### Definitions and outcomes

The diagnosis was based on codes from the International Classification of Diseases 10th revision (ICD-10). VSD was diagnosed when a child had ICD-10 codes (Q21.0, ventricular septal defect; Q21.00, muscular ventricular septal defect; Q21.01, perimembranous ventricular septal defect; Q21.08, other ventricular septal defect; and Q21.09, ventricular septal defect, unspecified).

The primary endpoint was hospital admission for CAP diagnosed when a child had ICD-10 codes from J12X to J18X from NHIS claims-based data of inpatients. CAP hospital admission was also analyzed when a child had ICD-10 codes from J12X to J18X for inpatients after 12 months. The VSD with HF was analyzed when a child had ICD-10 codes from I50X for inpatients after 12 months.

Asthma was diagnosed when a child had ICD-10 codes of J45X or J46X at least twice and a prescription history of asthma medication (inhalation steroid and anti-leukotriene) from NHIS claims-based data of outpatients or admission history with ICD-10 codes of J45X or J46X for inpatients. The secondary endpoint was hospital admission for asthma diagnosed with ICD-10 codes of J45X or J46X for inpatients after 24 months. Meanwhile, atopic dermatitis (AD) and allergic rhinitis (AR) were diagnosed when a child had ICD-10 codes of L20.9 and J30. 4, respectively, at least five times for outpatients from NHIS claims-based data of outpatients.

### Covariate and follow-up

Birthday, birth weight, prematurity, and breast milk feeding information were obtained from answers (by parents) to questionnaire items in the first round of the national health-screening program. Birth residence was used in the eligibility database divided into three regions (Seoul/metropolitan *vs*. urban *vs*. rural). Household income was determined based on insurance co-payment amount in eligibility database quintiles. Comorbidity allergic diseases including AD and AR were used in the eligibility database and divided into two classes (yes *vs*. no).

We separately analyzed subjects' primary (hospital admission for pneumonia) and secondary (hospital admission for asthma) endpoints. All participants were followed up from the index date (= birth date) until the diagnosis of pneumonia or asthma, outpatient visit, admission to the hospital, or the end of the study (December 31, 2019). To reduce risks of reverse causality and surveillance bias, we excluded those with the first year of follow-up for pneumonia and those with the first two years of follow-up for asthma. Follow-up of unexposed children was additionally censored if they were later diagnosed with VSD. These children were then moved to the exposed group.

### Statistical analysis

Continuous variables are expressed as mean ± standard deviation (SD) based on data normality. They were compared using an independent sample *t*-test or Mann–Whitney *U* test, as appropriate. Cumulative incidence of events at 10 years was calculated based on Kaplan–Meier censoring estimates. Clinical outcomes of two groups were compared with the log-rank test.

Primary analyze was performed after adjusting for confounding factors. First, a multivariable Cox regression model was used. Covariates were sex, birth weight (< 3.2 kg or ≥ 3.2 kg), age at index date (1–5 years or ≥ 5 years), calendar year at index date (2008–2009 or 2010–2012), allergic disease (AR, AD, and asthma), and comorbidity number of hospital admission with any reason or wheezing during the first year after study entry (yes or no). Second, to reduce selection bias and other potential confounding factors, we performed an analysis using the logistic regression model with an inverse probability of treatment weighting (IPTW) for sensitivity analyses^17^. Adjusted covariates in IPTW analysis included sex, birth weight, calendar year at birth date, birth residence, and income quintile.

We established a multivariable Cox proportional hazards model to identify independent predictors over a median follow-up of 7 years’ pneumonia and major adverse events. Primary end point comparison according to the various exploratory subgroups was then performed. In all analyses, participating centers were included as random effects. All statistical analyses were performed using R (version 3.1.1) and SAS software ver. 9.4 (SAS Institute, Cary, NC, USA).

### Institutional review board

The Korea National Institute for Bioethics Policy Institutional Review Board approved this study (P01-201603-21-005) due to retrospective nature.

## Results

### Baseline characteristics

A total of 103,400 children participated in this study, including 94,000 in the non-VSD group and 9400 in the VSD group. We first examined children's essential sociodemographic and clinical characteristics in VSD and non-VSD groups (Table 1). VSD and non-VSD groups had male gender in 46.26% and 51.23%, respectively. Among the VSD group, the two leading diagnosis types were Q21.09 (ventricular septal defect, unspecified, N = 2,967 (31.56%)) and Q21.0 (ventricular septal defect, N = 2,235 (23.78%)).

**Table 1:** Baseline characteristics of subjects.

Characteristics	VSD group	Non-VSD group
Number, No. (%)	9400 (100%)	94,000 (100%)
*Follow-up time, mean (SD), y*		
Pneumonia	6.67 (3.14)	6.96 (3.04)
Asthma	7.65 (2.26)	7.78(2.24)
No. of male (%)	4348 (46.26%)	48,157 (51.23%)
Birth weight, mean (SD), kg	3.2 (0.4)	3.2 (0.4)
No. of prematurity	241 (2.56%)	1400 (1.49%)
No. of only breast feeding	2404 (25.57%)	25,098 (26.70%)
*Birth residence, No. (%)*		
Seoul & metropolitan	4452 (47.36%)	42,321 (45.02%)
City	4173 (44.39%)	43,047 (45.79%)
Rural	689 (7.33%)	7557 (8.04%)
*Calendar year at birth date, No. (%)*		
2008–2009	3377 (35.93%)	36,741 (39.09%)
2010–2012	6023 (64.07%)	57,259 (60.91%)
*Income level, No. (%)*		
Lowest 20%	987 (10.5%)	10,170 (10.82%)
Middle	5611 (59.69%)	55,868 (59.43%)
Top 20%	2457 (26.14%)	24,106 (25.64%)
*Allergic disease comorbidity, No. (%)*		
Allergic rhinitis	2974 (31.55%)	28,365 (30.18%)
Atopic dermatitis	789 (8.39%)	8089 (8.61%)
*Admission during the first year after birth date*		
0 times	5152 (54.81%)	68,408 (72.77%)
≥ 1 times	4248 (45.19%)	25,592(27.23%)
*Admission with pneumonia during the first year*		
0 times	8689 (92.44%)	88,317 (93.95%)
≥ 1 times	711 (7.56%)	5683 (6.05%)
*Admission with wheezing during the first year*		
0 times	7808(83.06%)	84,032(89.40%)
≥ 1 times	1.592 (16.94%)	9968 (10.60%)
Diagnosis type, No. (%)	9400 (100.0%)	
Q21.0 Ventricular septal defect	2235 (23.78%)	NA
Q21.00 Muscular ventricular septal defect	1248 (13.28%)	NA
Q21.01 Perimemberance ventricular septal defect	1771 (18.84%)	NA
Q21.08 Other ventricular septal defect	1179 (12.54%)	NA
Q21.09 Ventricular septal defect, unspecific	2967 (31.56%)	NA

Data are presented as n (%) or mean, SD.

*SD* standard deviation.

The final assessment was on December 31, 2019. The median follow-up was 6.67 (3.14) years for those with pneumonia and 7.65 (2.26) years for those with asthma in the VSD group. It was 6.96 (3.04) year for those with pneumonia and 7.78 (2.24) years for those with asthma. Compared with the control group, children with VSD were more likely to be male portion (*P* < 0.001), prematurity (*P* < 0.001), only breast milk feeding (*P* = 0.019), residency of birth (*P* < 0.001), and birth date (*P* < 0.001). However, children with VSD were no more likely to be income level (*P* = 0.436) and allergic disease comorbidity (AR (*P* = 0.429) and AD (*P* = 0.497). The allergic disease comorbidity was found in 2,974 (31.55%) children with AR and 1,359 (14.46%) children with asthma in the VSD group (Table 1).

### Risk of CAP among children with VSD compared with matched non-VSD children

Results of comparing follow-up clinical outcomes of CAP between VSD and non-VSD groups are shown in Table 2. During a mean follow-up of 6.67 years (starting from 12 months after birth), we identified 2100 with a newly diagnosed CAP admission among exposed patients (incidence rate: 33.2 per 1000 person-years) and 20,109 CAP admission cases among unexposed children (incidence rate: 29.6 per 1000 person-years). This corresponded to an absolute rate difference of 3.65 (95% confidence interval (CI): 2.20–5.10) per 1000 person-years. After controlling for confounders, the risk for CAP during the second year after study entry was increased among patients with VSD (HR: 1.09, 95% CI 1.04–1.14) compared with matched children without VSD. Consistent results were found in IPTW analyses. After IPTW, the absolute rate difference was 2.96 (95% CI 1.42–4.49) per 1000 person-years.

**Table 2:** Risk of CAP among children with VSD compared with matched unexposed individuals (after 12 months, index date = birth date).

	No. of Pneumonia admission cases/No of Accumulated person-years X 1000 (incidence rate/1000 person-years)						Absolute rate difference/10,000person-years (95% CI)	Hazard Ratio (95% CI)*	Interaction *P* value**
	Control			VSD					
	Total	No. of pneumonia cases	Incidence rate/1000 person-years	Total	No. of pneumonia cases	Incidence rate/1000 person-years			
All	94,000	20,109	29.60	9400	2190	33.20	3.65(2.20–5.10)	1.09(1.04–1.14)	NA
All***	85,260	18,787	30.70	8526	2010	33.70	2.96 (1.42–4.49)	1.09(1.04–1.14)	NA
*Sex*									
Female	45,843	9712	29.2	5052	1159	32.4	3.27(1.32–5.23)	1.08(1.01–1.15)	0.564
Male	48,157	10,397	30.0	4348	1031	34.2	4.18(2.02–6.35)	1.11(1.04–1.18)	
*Birth weight, mean (SD), kg*									
< Median(3.2 kg)	36,720	8156	31.1	4150	999	34.9	3.83(1.56–6.09)	1.12(1.04–1.19)	0.486
≥ Median(3.2 kg)	51,038	11,195	30.5	4773	1109	32.8	2.32(0.31–4.34)	1.07(1.01–1.14)	
*Period since birth date, year*									
1 –5	9579	2927	43.4	997	320	47.6	4.21(–1.24–9.66)	1.07(0.95–1.21)	0.747
≥ 5	84,338	17,182	28.1	8375	1870	31.6	3.54(2.05–5.03)	1.09(1.04–1.15)	
*Calendar year at birth date, No. (%)*									
2008 – 2009	36,741	7981	25.6	3377	783	28.0	2.32(0.28–4.36)	1.07(0.99–1.16)	0.462
2010 – 2012	57,259	12,128	32.9	6023	1407	37.1	4.21(2.18–6.24)	1.11(1.04–1.17)	
*Residence*									
Seoul/Metropolitan	42,321	8576	27.5	4452	988	31.7	3.70(1.67–5.73)	1.09(1.02–1.17)	0.607
City	43,047	9196	29.9	4173	955	29.2	2.85(0.69–5.01)	1.07(1.00–1.15)	
Rural	7557	2115	40.9	689	226	4.4	10.08(3.20–16.95)	1.21(1.05–1.40)	
*Income level*									
Lowest 20%	10,170	2373	32.8	987	253	37.4	4.56(–0.23–9.35)	1.08(0.95–1.24)	0.608
Middle	55,868	12,378	30.8	5611	1371	35.1	4.31(2.38–6.25)	1.11(1.05–1.17)	
Top 20%	24,106	4489	25.2	2457	482	27.2	1.97(–0.57–4.50)	1.05(0.96–1.16)	
*Comorbidity-Asthma*									
No	80,466	15,869	26.9	8041	1753	30.8	3.82(2.32–5.32)	1.11(1.06–1.17)	0.169
Yes	13,534	4240	46.7	1359	437	49	2.31(–2.50–7.11)	1.02(0.92–1.13)	
*No. of admission during the second year after study entry, No. (%)*									
0 time	60,673	6115	12.6	4866	540	14.1	1.51(0.28–2.74)	1.09(0.99–1.19)	< .0001
≥ 1 times	33,327	13,994	72.1	4534	1650	59.7	− 12.36(–15.48––9.24)	0.84(0.79–0.88)	
*No. of admission with pneumonia during the second year after study entry, No. (%)*									
0 time	81,483	10,171	15.8	7972	1064	17.3	1.44(0.35–2.52)	1.06(0.99–1.13)	0.067
≥ 1 times	12,517	9938	262.1	1428	1126	259.4	− 2.7(–18.71–13.31)	0.97(0.91–1.03)	
*No. of outpatient visit during the second year after study entry, No. (%)*									
< median(= 56)	47,720	9128	25.5	4097	866	29	3.44(1.44–5.44)	1.12(1.04–1.20)	0.335
≥ median(= 56)	46,280	10,981	34.1	5,303	1,324	36.8	2.69(0.61–4.77)	1.05(0.99–1.12)	
*No. of admission with wheezing during the second year after study entry, No. (%)*									
0 time	76,938	10,879	18.3	7129	1072	19.8	1.52(0.29–2.75)	1.06(0.99–1.13)	< .0001
≥ 1 times	17,062	9230	110.2	2271	1118	95.7	− 14.49(–20.53––8.45)	0.87(0.81–0.93)	
*No. of admission in ICU during the second year after study entry, No. (%)*									
0 time	93,731	20,042	29.6	8055	1829	32.1	2.56(1.03–4.08)	1.06(1.01–1.12)	0.672
≥ 1 times	269	67	39.9	1345	361	40.3	0.45(− 9.96–10.87)	1.14(0.85–1.53)	

*CAP* community acquired pneumonia, *VSD* ventricle septal defect, *CI* confidence interval, *HR* hazard ratio, *ICU* intensive care unit, *NA* not applied.

* Cox models were adjusted for sex, birth weight, calendar year at birth date, birth residence, and income quintile. The first year of follow-up was excluded for all analyses.

** *P-*value was derived from interaction test by incorporating an interaction term to the Cox model.

***Inverse probability of treatment weighting-adjusted for sex, birth weight, calendar year at birth date, birth residence, and income quintile.

In subgroup analysis for follow-up period admissions, these associations were not stronger among children with VSD regarding the number of admissions during the second year after study entry (HR: 1.09 [95% CI 0.99–1.19] and 0.84 [95% CI 0.79–0.88] for 0 time and ≥ 1 time, respectively; *P* for interaction < 0.001) or the number of admission with wheezing during the second year after study entry (HR: 1.06 [95% CI 0.99–1.13] and 0.87 [95% CI 0.81–0.93] for 0 time and ≥ 1 time, respectively; *P* for interaction < 0.001) (Table 2).

**Figure 2:**
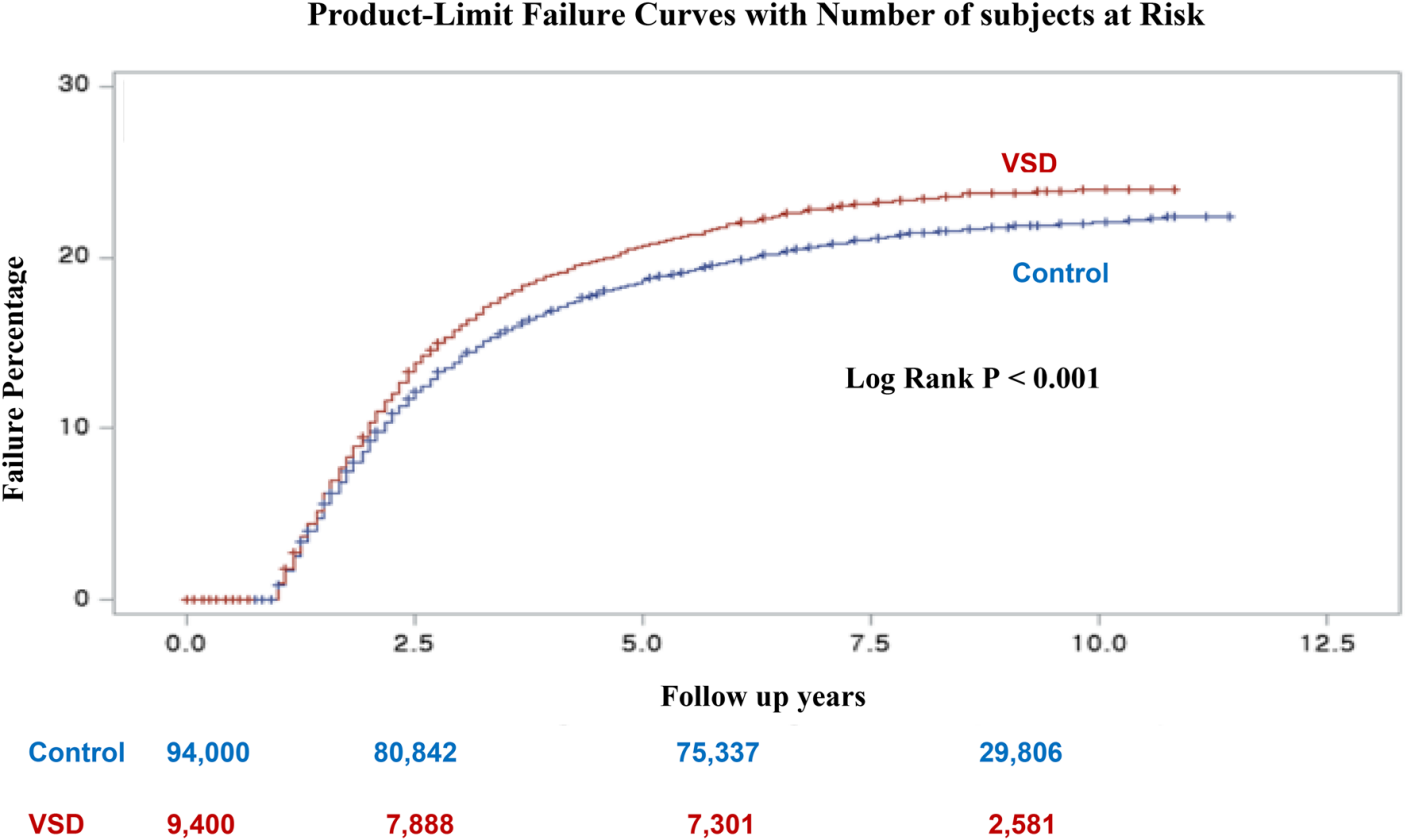
Kaplan–Meier curves for 10-year failure in patients with VSD children and matched non-VSD children. *VSD* ventricle septal defect.

After restricting participants to be more than five years of follow-up, children with VSD had a higher cumulative incidence of CAP than their matched non-VSD children across the follow-up period (log-rank test *P* < 0.001) (Fig. 2).

### Risk of CAP among children with VSD according to HF and operation

During a mean follow-up of 6.79 years (control), 6.27 years (VSD with no HF), and 3.26 years (VSD with HF) (starting from 12 months after birth), we identified 20,109 CAP admission cases among control (incidence rate: 29.6 per 1000 person-years) and 8887 CAP admission cases among VSD with no HF and 513 CAP admission cases among VSD with HF, respectively (Table 3). After controlling for confounders, the risk for CAP during the second year after study entry was increased among patients with VSD with no HF (HR: 1.08, 95% CI 1.03–1.13) and among patients with VSD with HF (HR: 1.42, 95% CI 1.20–1.68), compared with matched children without VSD. Also, the risk for CAP during the second year after study entry was increased in patients with VSD with HF than in patients with VSD with no HF (Table 3).

**Table 3:** Risk of CAP among children with VSD, according to HF and OP (after 12 months, index date = birth date).

	No. of Pneumonia admission cases/No of Accumulated person-years X 1000 (incidence rate/1000 person-years)			Absolute rate difference/10,000person-years (95% CI)	Hazard ratio *P* value** (95% CI)*				
	Total	No. of pneumonia cases	Incidence rate/1000 person-years						
Control	940,000	20,109	29.6	Ref	< 0.001				
VSD with no HF	8887	2043		0.02(0.01–0.03)	1.08(1.03–1.13)				
VSD with HF	513	147		0.07(0.04–0.11)	1.42(1.20–1.68)				

	No. of Pneumonia admission cases/No of Accumulated person-years X 1000 (incidence rate/1000 person-years)						Absolute rate difference/10,000 person-years (95% CI)	Hazard Ratio (95% CI)*	*P* value**
	Matched VSD non-OP			VSD-OP					
	Total	No. of pneumonia cases	Incidence rate/1000 person-years	Total	No. of pneumonia cases	Incidence rate/1000 person-years			
*Heart OP*									
Before	9137	181	67.3	263	12	0.1	0.03(0.01–0.04)	2.85(1.44–5.67)	0.003
After	9137	53.2	36.2	263	56	28.7	0.00(− 0.05–0.05)	1.00(0.75–1.33)	

*HF* heart failure, *OP* operation, *CAP* community acquired pneumonia, *VSD* ventricle septal defect, *CI* confidence interval; HR, hazard ratio.

* Cox models were adjusted for sex, birth weight, calendar year at birth date, birth residence, and income quintile. The first year of follow-up was excluded for all analyses.

** *P-*value was derived from interaction test by incorporating an interaction term to the Cox model.

In subgroup analysis for follow-up period admissions, these associations were stronger among children with VSD operation at the number of admissions during the second year after study entry (HR, 2.85 [95% CI 1.44–5.67] and 1.00 [95% CI 0.75–1.33] for before and after, respectively; *P* for interaction = 0.003) (Table 3).

### Risk of asthma among children with VSD compared with matched non-VSD children

Results of comparing follow-up clinical outcomes in asthma between VSD and non-VSD groups are shown in Table 4. During a mean follow-up of 7.65 years (starting from 24 months after birth), we identified 1010 asthma admission cases among exposed patients (incidence rate: 14.0 per 1000 person-years) and 9820 cases among unexposed children (incidence rate: 13.4 per 1000 person-years). This corresponded to an absolute rate difference of 6.25 (95% CI − 2.81–15.31) per 1000 person-years. After controlling for confounders, the risk for asthma admission or ER visit cases during the second year after study entry was increased among patients with VSD (HR: 1.05 [95% CI 0.99–1.13]) compared with matched children without VSD. Consistent results were also found in IPTW analyses, showing an absolute rate difference of 0.42(95% CI − 0.55–1.38) per 1000 person-years after IPTW.

**Table 4:** Risk of asthma among children with VSD compared with matched non-VSD children after 24 months (index date = birth date).

	No. of asthma cases/No of Accumulated person-years X 1000 (incidence rate/1000 person-years)						Absolute rate difference/10,000 person-years(95% CI)	Hazard Ratio(95% CI)*	Interaction *P* value**
	Control			VSD					
	Total	No. of asthma cases	Incidence rate/1000 person-years	Total	No. of asthma cases	Incidence rate/1000 person-years			
All	94,000	9820	134.2	9400	1010	140.5	6.25(− 2.81–15.31)	1.05(0.99–1.13)	NA
All***	85,260	9254	14.0	8526	940	14.4	0.42(− 0.55–1.38)	1.03(0.96–1.10)	NA
*Sex*									
Female	45,843	4361	121.2	5052	500	128.2	6.97(− 4.83–18.77)	1.06(0.96–1.16)	0.922
Male	48,157	5459	146.8	4348	510	155.0	8.23(− 5.78–22.23)	1.05(0.96–1.16)	
*Birth weight, mean (SD), kg*									
< median (value = 3.2)	36,720	4032	142.3	4150	449	142.5	0.21(− 13.68–14.11)	1.03(0.94–1.14)	0.565
≥ median	51,038	5411	136.6	4773	521	142.0	5.36(− 7.37–18.08)	1.07(0.98–1.18)	
*Period since birth date, year*									
1–5	9579	9523	3794.0	997	987	3796.2	2.13(− 246.66–250.92)	1.01(0.95–1.08)	0.403
≥ 5	84,338	297	4.2	8375	23	3.3	− 0.89(− 2.32–0.55)	0.88(0.57–1.36)	
*Calendar year at birth date, No. (%)*									
2008–2009	36,741	5196	158.3	3377	487	162.9	4.61(10.49–19.70)	1.02(0.93–1.13)	0.382
2010–2012	57,259	4624	114.7	6023	523	124.5	9.87(1.30–21.04)	1.09(0.99–1.19)	
*Residence*									
Seoul/Metropolitan	42,321	4262	127.8	4452	473	138.3	10.55(− 2.49–23.59)	1.10(1.00–1.21)	0.430
City	43,047	4667	141.3	4173	452	142.6	1.29(− 12.46–15.05)	1.01(0.91–1.12)	
Rural	7557	801	135.3	689	76	143.4	8.09(− 25.48–41.67)	1.07(0.83–1.38)	
*Income level*									
Lowest 20%	10,170	1077	136.2	987	107	140.8	4.63(− 23.26–32.52)	1.02(0.83–1.25)	0.409
Middle	55,868	5983	137.5	5611	622	145.0	7.48(− 4.44–19.40)	1.04(0.95–1.13)	
Top 20%	24,106	2364	125.9	2457	258	137.2	11.36(− 6.14–28.85)	1.11(0.98–1.27)	
*No. of admission during the second year after study entry, No. (%)*									
0 time	60,673	5647	118.1	4866	451	118.7	0.62(–10.76–12.00)	1.00(0.90–1.10)	0.574
≥ 1 times	33,327	4173	164.7	4534	559	164.9	0.22(− 14.34–14.77)	1.04(0.95–1.14)	
*No. of outpatient visit during the second year after study entry, No. (%)*									
< Median(= 56)	47,720	3926	102.1	4097	334	102.5	0.32(− 11.12–11.76)	0.99(0.88–1.11)	0.519
≥ Median(= 56)	46,280	5894	169.8	5303	676	172.0	2.25(− 11.42–15.92)	1.04(0.96–1.13)	
*No. of admission with wheezing during the second year after study entry, No. (%)*									
0 time	76,938	7346	121.4	7129	712	128.8	7.37(− 2.49–17.23)	1.07(0.98–1.15)	0.105
≥ 1 times	17,062	2474	195.7	2271	298	178.4	− 17.29(− 38.96–4.39)	0.95(0.83–1.07)	
*No. of admission in ICU during the second year after study entry, No. (%)*									
0 time	93,731	9786	134.1	8055	866	140.1	6.04(− 3.67–15.74)	1.05(0.98–1.13)	0.365
≥ 1 times	269	34	188.9	1345	144	142.6	− 46.32(− 113.94–21.31)	0.92(0.61–1.38)	
*Comorbidity-AR*									
No	65,635	5436	104.56	6426	539	107.58	3.03(− 6.47–12.53)	1.06(0.96–1.16)	0.549
Yes	28,365	484	207.09	2974	471	216.06	8.97(− 11.48–29.42)	1.01(0.92–1.12)	
*Comorbidity-AD*									
No	85,911	8620	128.87	8611	888	134.95	6.09(− 3.20–15.37)	1.03(0.96–1.10)	0.758
Yes	8089	1200	191.39	789	122	200	8.61(− 28.49–45.72)	1.06(0.88–1.29)	

*VSD* ventricle septal defect, *CI* confidence interval, *NA* not applied.

* Cox models were adjusted for sex, birth weight, calendar year at birth date, birth residence, and income quintile. First two years of follow-up were excluded for all analyses.

** *P* value was derived from interaction test by incorporating an interaction term to the Cox model.

***Inverse probability of treatment weighting-adjusted for sex, birth weight, calendar year at birth date, birth residence, and income quintile.

In subgroup analysis for follow-up period admissions, these associations were not stronger among children with VSD at the number of admissions during the second year after study entry (HR, 1.00[95% CI 0.90–1.10] and 1.04[95% CI 0.95–1.14] for 0 and ≥ 1 time, respectively; *P* for interaction > 0.05) or the number of admission with wheezing during the second year after study entry (HR, 1.07[95% CI 0.98–1.15] and 0.95[95% CI 0.83–1.07] for 0 and ≥ 1 time, respectively; *P* for interaction > 0.05) (Table 4).

## Discussion

In the present study, we investigated the association of VSD with CAP or asthma hospitalization in children with or without VSD in a large-scale, population-based nationwide pediatric cohort from Korea (n = 103,400). At the beginning of this study, we assumed that VSD might have some associations with the severity of CAP and asthma in the pediatric population during childhood. Thus, the current study had the following principal findings: (1) 12,378 (5.47%) individuals received their first diagnosis of VSD between birth and December 31, 2019, and (2) children with VSD were at elevated risk of hospital admission for CAP but not asthma. Moreover, using population-based comparisons, this is the first study to address VSD and its associations with CAP and asthma in the pediatric population.

CHD is a gross structural abnormality of the heart or intra thoracic great vessels that is actually or potentially of functional significance^6,7,18^. The incidence of CHD in the general population is about 1%, ranging from 4/1000 to 50/1000 live births^18^. In childhood, VSD, patent ductus arteriosus (PDA), atrioventricular septal defect (AVSD), and atrial septal defect (ASD) are common acyanotic CHD^6,18^. The incidence of isolated VSD is about 0.3% of newborns^6^, but the incidence is significantly lower in adults because > 90% may eventually close spontaneously^3^. The current study's prevalence of VSD (5.47%) was higher than in previous studies in the newborn registry^6^ and under five years of age^18^ because the study period was from birth to December 31, 2019, a short time to close spontaneously.

This study showed that children with VSD were at elevated risk of hospital admission for CAP but not asthma. CAP is the most common cause of death in children under five years old and is responsible for approximately 1.5 million ambulatory visits in children annually in the US ^1,19,20^. The leading cause of CAP is viral infection ^1,19,20^, and due to improvements in hygiene and successful vaccination programs, recent Korean statistics indicate that CAP is no longer the leading cause of death among children^21^.

There is pulmonary overcirculation and pulmonary edema^12^ because of left to right shunting of blood in children with CHD, including VSD. Due to this mechanism, patients with CHD have a higher risk for complications with viral diseases^4,5^. It is well-known that influenza infections are associated with high autopsy-confirmed coronary deaths among respiratory infections^3^. Additionally, in the case‐crossover study from a population‐based cohort^4^, CHD and ischemic stroke risk is higher after both in‐ and outpatient infection. Adults with infection had higher odds of CHD and ischemic stroke up to 90 days after infections compared with equivalent control periods 1 and 2 years before the event^4^. Recurrent acute lower respiratory tract infection often occurs in children with CHD^18^. Also, some CHD may predispose their sufferers to bronchopneumonia^8,9,11^, and children with pneumonia and CHD stayed significantly longer in the hospital than those without CHD. These results agreed with the present study, showing that children with VSD were at elevated risk of hospital admission for pneumonia compared to non-VSD children, and children with VSD with HF were at elevated risk of hospital admission for pneumonia compared to VSD children with non-HF. Therefore, children with VSD with HF might be at high risk because of their limited cardiopulmonary reserve.

Meanwhile, asthma in children is a significant concern because it increases the number of hospital visits and economic burden more than asthma in adults^22^. In the present study, we found that children with VSD were not significantly more frequently admitting asthma than non-VSD. This disagreement might be because asthma results from complex gene-environment interactions with heterogeneity in clinical presentation^23,24^. One study has demonstrated a high prevalence of airway hyperresponsiveness in patients with ASD, suggesting that airway hyperresponsiveness might be a possible mechanism for recurrent attacks of cough variant asthma^25^. The mechanism for cough variant asthma caused by heart disease is bronchial vascular congestion, resulting in bronchial edema and thickening. Furthermore, left ventricular dysfunction can lead to abnormal pulmonary function, such as airway hyperresponsiveness or restrictive and obstructive dysfunction^25^. A retrospective cohort study of children has documented that the hazard of recurrent cough variant asthma is higher in children with CHD, especially in children with complex congenital heart disease^26^. However, these two reports had no data about the relationship between VSD and asthma or data showing that CHD had how many diagnoses^25,26^.

The greatest strength of this study was that we performed various assessments of risk for VSD with CAP or asthma in a large population of children. However, this study has several limitations. First, children in our study were 11 years or younger. Thus, the generalizability of our findings to older children needs more long-term follow-up, and there may be differential misclassification where some children in the non-VSD group may have undiagnosed VSD, and the opposite is less likely. Second, because this was an observational study, measurement errors or misunderstandings of adjustment factors might have occurred. Additionally, surveillance bias could exist in this study. Third, no comparison exists between the influence of pre-and post-operative VSD on CAP or asthma hospitalization.

## Conclusions

In summary, evidence supports that childhood VSD is related to a higher surge in admission rates in children with CAP, not asthma, compared with matched children without VSD. Children with VSD might be at high risk of admission because of their limited cardiopulmonary reserve and thus need more intensive care to prevent viral infection. Further studies are needed to better understand the underlying mechanisms.

## Data Availability

Data is provided within the manuscript information files.
