# Author Correction: Population-based retrospective cohort study on community-acquired pneumonia hospitalization in children with a ventricular septal defect

**DOI:** 10.1038/s41598-024-61472-x

**Published:** 2024-05-10

**Authors:** Myongsoon Sung, Ju Hee Kim, Eun Kyo Ha, Jeewon Shin, Ji Hee Kwak, Hye Mi Jee, Man Yong Han

**Affiliations:** 1 Department of Pediatrics, Soonchunhyang University Gumi Hospital, Gumi, Republic of Korea; 2 Department of Pediatrics, National Jewish Health, Denver, CO USA; 3 Department of Pediatrics, Kyung Hee University College of Medicine, Seoul, Korea; 4 Department of Pediatrics, Hallym University Kangnam Sacred Heart Hospital, Seoul, Korea; 5 Department of Pediatrics, CHA Bundang Medical Center, CHA University School of Medicine, 351 Yatap-Dong, Bundang-Gu, Seongnam, 463-712 Gyonggi-Do Korea; 6 Department of Pediatrics, Kangbuk Samsung Hospital, Sungkyunkwan University School of Medicine, Seoul, Korea

Correction to: *Scientific Reports* 10.1038/s41598-024-59510-9, published online 23 April 2024

The original version of this Article contained an error in the name of Eun Kyo Ha, which was incorrectly given as Eun Gyo Ha.

The original Article has been corrected.

